# Supporting Informed Decision Making for Prostate Specific Antigen (PSA) Testing on the Web: An Online Randomized Controlled Trial

**DOI:** 10.2196/jmir.1305

**Published:** 2010-08-06

**Authors:** Rhodri Evans, Natalie Joseph-Williams, Adrian Edwards, Robert G Newcombe, Patricia Wright, Paul Kinnersley, Jeff Griffiths, Mari Jones, Janet Williams, Richard Grol, Glyn Elwyn

**Affiliations:** ^4^IQ Scientific Institute for Quality in HealthcareRadboud UniversityNijmegenNetherlands; ^3^School of MathematicsCardiff UniversityCardiffUnited Kingdom; ^2^School of PsychologyCardiff UniversityCardiffUnited Kingdom; ^1^Department of Primary Care and Public HealthSchool of MedicineCardiff UniversityCardiffUnited Kingdom

**Keywords:** Decision aid, Informed decision making, Internet, Prostate cancer, Prostate Specific Antigen (PSA) test

## Abstract

**Background:**

Men considering the prostate specific antigen (PSA) test for prostate cancer, an increasingly common male cancer, are encouraged to make informed decisions, as the test is limited in its accuracy and the natural history of the condition is poorly understood. The Web-based PSA decision aid, Prosdex*,* was developed as part of the UK Prostate Cancer Risk Management Programme in order to help men make such informed decisions.

**Objectives:**

The aim of this study was to evaluate the effect of the Web-based PSA decision aid, Prosdex*,* on informed decision making.

**Methods:**

A Web-based randomized controlled trial was conducted in South Wales, United Kingdom. Men aged 50 to 75 who had not previously had a PSA test were randomly allocated to two intervention and two control groups. Participants in the intervention groups either viewed Prosdex or were given a paper version of the text. The main outcome measures were the three components of informed decision making: (1) knowledge of prostate cancer and PSA, (2) attitude toward PSA testing, (3) behavior using a proxy measure, intention to undergo PSA testing. Decisional conflict and anxiety were also measured as was uptake of the PSA test. Outcomes were measured by means of an online questionnaire for the Prosdex group, the paper version group, and one of two control groups. Six months later, PSA test uptake was ascertained from general practitioners’ records, and the online questionnaire was repeated. Results are reported in terms of the Mann-Whitney U-statistic divided by the product of the two sample sizes (U/mm), line of no effect 0.50.

**Results:**

Participants were 514 men. Compared with the control group that completed the initial online questionnaire, men in the Prosdex group had increased knowledge about the PSA test and prostate cancer (U/mn 0.70; 95% CI 0.62 - 0.76); less favourable attitudes to PSA testing (U/mn 0.39, 95% CI 0.31 - 0.47); were less likely to undergo PSA testing (U/mn 0.40, 95% CI 0.32 - 0.48); and had less decisional conflict (U/mn 0.32, 95% CI 0.25 - 0.40); while anxiety level did not differ (U/mn 0.50, 95% CI 0.42 - 0.58). For these outcomes there were no significant differences between men in the Prosdex group and the paper version group. However, in the Prosdex group, increased knowledge was associated with a less favourable attitude toward testing (Spearman rank correlation [ρ] = -0.49, *P* < .001) and lower intention to undergo testing (ρ = -0.27, *P* = .02). After six months, PSA test uptake was lower in the Prosdex group than in the paper version and the questionnaire control group (*P* = .014). Test uptake was also lower in the control group that did not complete a questionnaire than in the control group that did, suggesting a possible Hawthorne effect of the questionnaire in favour of PSA testing.

**Conclusions:**

Exposure to Prosdex was associated with improved knowledge about the PSA test and prostate cancer. Men who had a high level of knowledge had a less favourable attitude toward and were less likely to undergo PSA testing. Prosdex appears to promote informed decision making regarding the PSA test.

**Trial Registration:**

ISRCTN48473735; http://www.controlled-trials.com/ISRCTN48473735 (Archived by WebCite at http://www.webcitation.org/5r1TLQ5nK)

## Introduction

Informed decision making is difficult to deliver for men who are considering the prostate specific antigen (PSA) test. Web-based PSA decision aids potentially provide a solution.

The decision to undergo PSA testing for prostate cancer is a very difficult one for men. On the one hand, the disease is gaining prominence, largely due to an aging population and increased detection. On the other hand, the usefulness of the only widely available test for prostate cancer, PSA, is limited not only by its poor sensitivity and specificity but also by the uncertainty relating to the natural history and the management of the disease [1,2]. It is for this reason that the UK National Cancer Screening Programme in 2001 established the Prostate Cancer Risk Management Programme (PCRMP), a strategy that had, as one of its key goals, the promotion of informed decision making about PSA testing [2]. An informed decision, as outlined in the measure of informed decision making developed by Marteau et al for prenatal Down’s syndrome testing [3,4], could be characterized by relevant knowledge of the subject as well as by attitudes congruent with subsequent behavior. In the case of PSA testing, therefore, what was important was not the actual decision made vis-à-vis the test. Instead, the aim of the PCRMP was for men to both acquire knowledge about PSA and prostate cancer and make decisions about the test that reflected their attitudes toward it.

In order to facilitate informed decision making, all general practitioners (GPs) in the United Kingdom were provided with information leaflets about PSA testing to be provided to patients on request. Nonetheless, it was accepted by policymakers that a more publicly engaging source of information was required. Consequently, attention turned to decision aids, which already had a strong record in facilitating difficult decisions across a range of conditions including prostate cancer [5]. In addition, the Web was identified as a suitable medium for delivering these decision aids to UK men. Therefore, the Web-based PSA decision aid, Prosdex [6], was commissioned by Cancer Research UK and launched in 2004. It was, and remains, an innovative intervention in eHealth, combining text and multimedia features with deliberation tools designed to directly promote informed decision making, reflecting the standards set by the International Patient Decision Aid Collaboration [7].

It is not known whether PSA decision aids have an effect on informed decision making. Systematic reviews have consistently shown these aids improve knowledge about PSA and prostate cancer, which is the initial, essential component of informed decision making [5,8-10]. In addition, PSA decision aids have been shown to affect behavior, that is, use of these aids typically results in a reduced likelihood of PSA testing [8,9]. However, to date there is no evidence that such conservative behavior is associated with greater knowledge of prostate cancer and PSA testing and with negative attitudes toward the test, as would be expected with informed decision making. 

There is no specific measure of informed decision making in PSA testing, and therefore, for this study, a composite evaluation was used based on three key components: knowledge, attitude, and behavior. Knowledge about PSA and prostate cancer was taken as the principal outcome for the study due to its key role in informed decision making: without knowledge, informed decision making cannot take place. The behavior component assessed was intention to undertake PSA testing, and this was measured at the same time as the attitude toward the PSA test. Crucially, to determine an effect on informed decision making, correlations between the knowledge, attitude, and behavior outcomes were examined. Our hypothesis was that, at group level, men using the Prosdex Web-based PSA decision aid would improve their knowledge and thereby develop both a less favorable attitude toward the test and a reduced intention to undergo testing, principally because increased knowledge would lead to an understanding of the uncertain benefits of the PSA test. Finally, six months later, we evaluated a second behavioral outcome, PSA test uptake. The hypothesis here, for the same reasons, was that PSA test uptake would be reduced in those men who had demonstrated informed decision making.

The potential of the Web was exploited in two ways in this study. First, as described, the decision aid was hosted on the Web. Second, an online research methodology was employed, namely, a four-armed randomized controlled trial with two intervention and two control groups, which allowed a comparison between Prosdex and a control group, between Web-based Prosdex and a paper version of the same information, and, between the two control groups in order to consider the Hawthorne effect of an outcome questionnaire. Our expectation was that participants presented with the questionnaire would be more likely to undertake PSA testing due to an increased awareness of the subject and exposure to popular opinions and media coverage, which tend to be uncritically positive about the benefits of PSA testing. The aim of this study, therefore, was to evaluate the effect of a Web-based PSA decision aid, Prosdex*,* on knowledge, attitudes and behavior—the components of informed decision making—using a Web-based questionnaire.

## Methods

### Study Design

A Web-based randomized controlled trial (RCT) was designed, composed of four groups: two intervention groups and two control groups.

### Setting

Men were invited to participate by their general practitioners (GPs) in South Wales, United Kingdom.

### Participants

#### Inclusion Criteria

Men aged 50 to 75 were invited to participate. This is the age range in which men typically request or are offered the PSA test. The participants accessed the study via the Internet and had to be able to use a computer, and they were asked to indicate this on the consent form. Those unable to participate due to inability to use a computer and those who did not respond to the invitation were counted separately, in line with CONSORT guidelines [11].

#### Exclusion Criteria

Participants who could not read English were excluded, as were those whose general practice records indicated that they had previously had prostate cancer or a PSA test.

#### Recruitment Process

Potential participants were identified from electronic patient registers. A staff member from each practice generated a list of potential participants aged 50 to 75 who had not had a PSA test or prostate cancer. A random sample of 100 men was selected from the list. A staff member with knowledge of the patients—usually a GP—was asked to screen out men who were unsuitable for the trial due to serious ill-health, specifically a terminal illness, dementia, or severe mental illness. Finally, invitation letters signed by the GP, participant information sheets, and consent forms were sent by mail to eligible potential participants.

Affirmative consent forms from each practice were transferred to the research officer (author NJ-W) who allocated each participant with a number provided remotely by the trial statistician (author RN) to ensure concealment. The process ensured individual level randomization to one of two intervention groups or to one of two control groups, as we were interested in individual decision making after an intervention used independently by each participant. There was unlikely to be an intracluster correlation for these outcomes, because men would view the Web at home [12].

#### Sample Size

The sample size aimed for was 600 participants: 150 in each of the four groups. This figure was derived from our systematic review of PSA decision aids in which these interventions were found to have resulted in knowledge that had improved by 19.5% (SD 45.1) [9]. Thus, 150 men per group would allow the detection of a 20% absolute difference in knowledge with power greater than 90%. Assuming a recruitment rate of 30%, we aimed to invite 2000 potential participants from 20 GP practices, 100 men from each practice [13].

### Intervention and Controls

**Figure 1:**
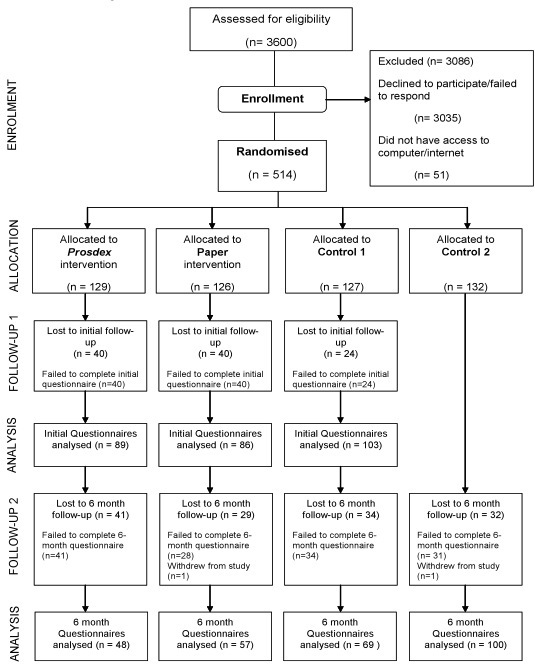
Prosdex RCT CONSORT participant flow chart

The intervention used in this study, the Web-based PSA decision aid Prosdex [6], presents evidence-based information about prostate cancer and PSA testing, encouraging users to weigh the pros and cons of testing [14]. In addition, Prosdex includes video clips of enacted patient experiences about the PSA test and subsequent investigations and treatments. There is also information about shared decision making and a deliberation tool named a “decision stacker,” which visualizes attitudes toward the PSA test. Prosdex aims to actively encourage informed decision making. A specific version of the Prosdex website was developed for the study. Participants in the Prosdex intervention group were able to view the intervention in their own homes or in other settings. Participants in the second intervention, the paper version group, received a paper document, comprising the text of the website but not the name, to reduce the risk of participants in this group discovering Prosdex on the Web. Participants in the control groups received neither the Prosdex URL nor the paper version of the website (Figure 1).

### Outcomes

Components of informed decision making were evaluated. Components were: (1) knowledge of prostate cancer and PSA, the primary outcome of the study; (2) attitude toward PSA testing; and (3) behavior, using the proxy measure of intention to undergo PSA testing.

Knowledge, attitude, and intention were assessed by a set of questions previously used in an evaluation of a brief paper-based PSA decision aid [15]. Twelve true or false questions were posed, and participants received a score of 1 for a correct answer, -1 for an incorrect answer, and 0 for unanswered items. In the attitude section, twelve statements, six favorable and six unfavorable to PSA testing, were presented. For the intention outcome, a single item question with a five-point Likert-like response scale, “How likely are you to have the PSA test?” was used, and a mean rank score was then calculated for each group.

Two other outcomes, anxiety and decisional conflict, were measured by the questionnaire as these are outcomes that are commonly used in evaluations of decision aids [9]. Anxiety was assessed using the six-item short form of the Spielberger State Anxiety Inventory [16,17], and decisional conflict was measured by the Decisional Conflict Scale (DCS) [18,19]. Finally, at 6-months post intervention, actual uptake of the PSA test was measured.

### Measurement of Outcomes

#### Baseline Outcomes Immediately Following the Intervention

Data for all outcomes, apart from test uptake, were gathered from responses to an online questionnaire. Participants allocated to the two intervention groups (the Prosdex group and the paper version group) and the first control group (named the “questionnaire control group”) were asked to complete a questionnaire. These 3 groups were asked to access the study website using a unique password that was provided them. Participants’ exposure to interventions and the questionnaire is shown in Figure 1, as is the pathway for participants in the second control group. The second control group, which was not asked to complete a questionnaire, was named the “no questionnaire control group.” The data were collected in an SQL-server database, transferred to Excel spreadsheets, and analyzed using SPSS, version 12 (SPSS Inc, Chicago, IL, USA).

#### Outcomes at Six Months

At 6 months after the participants were allocated to the four groups, GPs were asked to review participants’ medical records to ascertain whether participants had had a PSA test in the 6-month interval. All participants, including those in the no questionnaire control group, were also asked to repeat the online questionnaire in order to evaluate any changes to the baseline outcomes, such as knowledge, over a 6-month period.

### Comparisons and Statistical Analysis

To determine the effect on informed decision making, the main comparison was Prosdex group versus questionnaire control group. There were also two other comparisons. The first was the Prosdex group versus the paper version group to compare the effect of format and media. Also compared were the questionnaire control group versus the no questionnaire control group to assess the Hawthorne effect of the questionnaire on PSA testing as our expectation was that answering the questionnaire itself would encourage PSA testing. Also, in order to further assess the effect on informed decision making, correlations between knowledge, attitudes, and behavior outcomes were examined.

Most of the analyses in this study were undertaken on a group basis. However, according to the model of informed decision making, we should be able to demonstrate high knowledge, negative attitude/intention, and reduced uptake of PSA testing at the level of an individual participant as evidence of informed decision making by the individual. The data were therefore analyzed accordingly, and participants whose knowledge scores were above the median were defined as “high knowledge.” The attitude and intention outcomes were then also dichotomized on the basis of the median scores to “high” and “low.” Finally, the PSA test uptake data for individuals were analyzed in relation to the dichotomized outcomes, and the odds ratios for test uptake were calculated.

Comparability of the four groups for characteristics of age, ethnicity, marital status, and education was assessed. Demographic data for the two intervention groups and the questionnaire control group were obtained from the baseline questionnaire, and demographic data for the no questionnaire control group was obtained from the 6-month follow-up questionnaire. Outcomes were compared between groups on an ”intention to treat” basis. Results were reported in terms of an effect size derived from the Mann-Whitney U-statistic divided by the product of the two samples sizes (U/mn) [20]. The statistic U/mn is applicable to continuous and ordinal outcomes alike. The expected value under the null hypothesis is 0.5, and the measure takes the value 0 or 1 in extreme cases in which there is no overlap between the two samples. Therefore, for comparisons, U/mn is greater than 0.5 when the first group scores higher than the first; also, for confidence intervals, the line of no effect is set at 0.5 [20].

The study received ethical approval from South East Wales Research Ethics Committee (REC reference number 06/WSE04/13). All participants gave informed consent before taking part in the trial.

## Results

### Recruitment of General Practices

We invited 60 general practices from 9 Local Health Board (primary care organization) areas in South Wales. Of these, 27 practices agreed to participate, 33 declined, and 2 subsequently withdrew.

### Recruitment of Participants

In 2008, 25 practices were sent 100 invitations each, and in response to lower than anticipated recruitment, 11 of these practices agreed to send an additional 100 invitations. A total of 3600 invitations were sent. Consent forms were returned from 646 potential participants (18%), of whom 565 (16%) agreed to participate. Excluded were 51 participants due to reported difficulties with Internet access and/or computer literacy, giving a final number of 514 participants (Figure 1)*.* Of the 382 participants allocated to the Prosdex group, the paper version group, and the questionnaire control group, 278 (73%) completed the first online questionnaire in 2008. For the comparison of the Prosdex group versus the questionnaire control group, 89 versus 103 participants, a power of 87% was achieved to detect a 20% improvement in knowledge. For the Prosdex group versus the paper version group, 89 versus 86 participants, a power of 85% was achieved to detect a 20% improvement in knowledge.

### Characteristics of Participants

Details of participant characteristics are given in Table 1. Of the participating men, 236 of 379 (62%), were between 50 and 59 years of age, 367 (97.4%) were white, 79 (88%) were married or cohabitating, and 334 (45%) had either a college or university degree, indicating volunteer bias towards those who are younger and those with higher educational attainments. There were no statistically significant differences between the groups.

**Table 1:** Characteristics of trial participants

Characteristic		Prosdex Group n (%)	Paper Version Group n (%)	Questionnaire Control Group n (%)	No Questionnaire Control Group n (%)	Total n (%)
**Age group**						
	50-59	61 (68)	54 (63)	63 (61)	58 (58)	236 (62)
	60-69	22 (24)	27 (31)	35 (34)	36 (36)	120 (32)
	70 or over	7 (8)	5 (6)	5 (5)	6 (6)	23 (6)
**Highest level of education**						
	None	9 (10)	9 (11)	12 (12)	14 (14)	44 (12)
	CSE or equivalent	7 (8)	3 (4)	4 (4)	5 (5)	19 (5)
	O level or equivalent	10 (11)	16 (19)	14 (14)	13 (13)	53 (14)
	A level or equivalent	9 (10)	8 (9)	10 (10)	12 (12)	39 (10)
	Clerical or commercial	7 (8)	18 (21)	22 (21)	8 (8)	55 (15)
	College or university degree	48 (53)	32 (37)	41 (40)	48 (48)	169 (45)
**Marital status**						
	Married or cohabiting	79 (88)	76 (88)	90 (87)	89 (89)	334 (88)
	Widowed	1 (1)	0 (0)	4 (4)	0 (0)	5 (1)
	Never married	3 (3)	5 (6)	4 (4)	6 (6)	18 (5)
	Divorced or separated	7 (8)	5 (6)	5 (5)	5 (5)	22 (6)
**Ethnicity**						
	White	89 (99)	83 (98)	97 (95)	98 (99)	367 (98)
	Black African	0 (0)	0 (0)	1 (1)	0 (0)	1 (3)
	Indian	0 (0)	0 (0)	1 (1)	0 (0)	1 (3)
	Mixed race	1 (1)	2 (2)	1 (1)	1 (1)	5 (1)
	Other	0 (0)	0 (0)	2 (2)	0 (0)	2 (5)

### Comparisons and Correlations at Baseline

The two sets of comparisons at baseline, the Prosdex group versus the questionnaire control group and the Prosdex group versus the paper version group, are demonstrated in Table 2 with U/mn values. Results shown in Table 2 are based on 278 to 283 participants for whom full data were available. For the main comparison, the Prosdex group versus the questionnaire control group, participants in the Prosdex group were found to have significantly higher knowledge scores than those in the questionnaire control group. Men in the Prosdex group also had less favorable attitudes to the PSA test than those in the questionnaire control group, and intention to undergo PSA testing was lower in the Prosdex group than in the questionnaire control group. The decisional conflict score was lower in the Prosdex group than in the questionnaire control group, and there was no statistical difference between the two groups in terms of their anxiety regarding the PSA test.

Regarding the Prosdex group versus the paper version group, there was no significant difference in the knowledge scores of the participants. However, the mean knowledge score was slightly higher in the paper version group than in the Prosdex group (5.4 out of 12 vs 4.9 out of 12). Also, no significant differences were found between the Prosdex and the paper version groups in attitude toward PSA testing, intention to undergo PSA testing, decisional conflict score, and anxiety outcomes.

**Table 2:** Summary of outcome results

Outcome Measure	Prosdex Group vs Questionnaire Control Group				Prosdex Group vs Paper Version Group			
	Mean scores	U/mn	95% CI	*P* value	Mean scores	U/mn	95% CI	*P* value
Knowledge	4.90 vs 2.17	0.70	0.62 - 0.76	<.001	4.90 vs 5.40	0.47	0.39 - 0.55	.48
Attitude	9.10 vs 11.90	0.39	0.31 - 0.47	.007	9.10 vs 8.48	0.54	0.45 - 0.62	.39
Intention to undergo PSA testing^a^	40% vs 58%	0.40	0.32 - 0.48	.02	40% vs 53%	0.43	0.35 - 0.51	.10
DCS	40.37 vs 47.73	0.32	0.25 - 0.40	<.001	40.37 vs 38.49	0.56	0.47 - 0.64	.18
Anxiety	4.98 vs 4.88	0.50	0.42 - 0.58	.98	4.98 vs 4.78	0.51	0.43 - 0.60	.74

^a^ Figures reported for these cells are percentages of men who indicated they were very likely to or definitely would take the test.

The correlations between outcomes are shown in Table 3. There was a substantial inverse correlation between overall knowledge and attitude scores in the two intervention groups but not in the questionnaire control group. That is, greater knowledge was associated with a less favorable attitude toward the test in the two intervention groups. There was also a negative correlation between knowledge and intention to undergo PSA testing in the Prosdex group but not in the other two groups. In other words, for participants in the Prosdex group alone, greater knowledge was associated with reduced intention to take the PSA test. For all three groups, attitude and intention outcomes were strongly correlated. Therefore, as hypothesized at the outset of the study, participants in the Prosdex group demonstrated greater knowledge and had negative but congruent attitudes and behavior.

**Table 3:** Correlation of knowledge, attitude, and intention outcomes for the Prosdex, paper version, and questionnaire control groups

Outcome		Intervention Group
		Prosdex Group (n = 89)	Paper Version Group (n = 86)	Questionnaire Control Group (n = 103)
**Knowledge-Attitude**				
	Spearman rank correlation	-0.49	-0.49	-0.03
	*P* value^a^	< .001	< .001	.78
**Knowledge-Intention**				
	Spearman rank correlation	-0.27	-0.10	-0.02
	*P* value^a^	.01	.38	.87
**Attitude-Intention**				
	Spearman rank correlation	0.68	0.54	0.61
	*P* value^a^	< .001	< .001	< .001

^a^ 2-tailed significance

#### Comparisons at 6-month Follow-up

Although the trial design was not powered to demonstrate a difference at the level of PSA test uptake, there was a difference between the groups which achieved statistical significance. As shown in Table 4, PSA uptake by men in the Prosdex group was 3% (4/127) and in the questionnaire control group was 9% (11/123), *P* = .014. The Hawthorne effect of the questionnaire was also demonstrated, as the PSA test uptake in the no questionnaire control group was 2% (2/126), that is, less than that in the questionnaire control group.

In Table 5 we demonstrate the odds ratio of PSA testing, for individual participants, with respect to dichotomized knowledge/attitude/intention. PSA testing was found to be increased twofold for individuals with high attitude and high intention in the presence of high knowledge, although the confidence intervals were very wide. Finally, at six months, as shown in Table 6, knowledge was found to be significantly lower in both intervention groups but particularly so in the paper version group.

**Table 4:** Percent PSA test uptake by group

	Intervention and Control Groups (Percent Uptake)				
PSA Test Uptake^a^	Prosdex Group	Paper Version Group	Questionnaire Control Group	No Questionnaire Control Group	All Groups
No PSA test	96.9	90.9	91.1	98.4	94.4
PSA test	3.1	9.1	8.9	1.6	5.6

^a^ Pearson chi-square: *P* = .014

**Table 5:** Odds ratios showing the relationship of PSA test uptake to attitude and intention scores, dichotomized at their medians

Dichotomized Outcome	Odds Ratio^a^	95% Confidence Interval
Attitude	1.80	0.5 - 6.3
Intention	1.41	0.6 - 7.7

^a^ Based on 131 men with knowledge scores above the median on knowledge, and below the median on attitude and intention

**Table 6:** Mean knowledge scores at baseline and at 6 months by group

		Intervention and Control Groups^a^			
Timing of Questionnaire		Prosdex Group (n = 47)	Paper Version Group n = 57)	Questionnaire Control (n = 69)	No Questionnaire Control ( n = 100)
**Baseline**					
	Mean knowledge score^b^	5.13	5.79	2.30	-
**6 months**					
	Mean knowledge score^b^	3.70	3.96	2.80	1.75

^a^ Restricted to 273 men with full data available at both assessment points

^b^ Knowledge scale ranges from -12 to +12

## Discussion

### Summary of Main Findings

The Web-based decision aid was found to promote informed decision making about the PSA test. It increased knowledge about the PSA test and prostate cancer, generated less favorable attitudes to PSA testing, and lessened the intention to undergo testing. Participants who used Prosdex and who demonstrated a high level of knowledge had less favorable attitudes and lower intentions to undergo PSA testing. This congruence between attitude and intention in the context of higher knowledge accords with our definition of informed decision making in PSA testing.

PSA test uptake was less in the no questionnaire control group than in the questionnaire control group, confirming the Hawthorne effect of increased testing in participants presented with the questionnaire. PSA test uptake was found to be lower in the Prosdex group than in the paper version group and the questionnaire control group, suggesting that, in effect, exposure to Prosdex counters the Hawthorne effect of participation, so that PSA uptake is more likely to return to background levels. Finally, the data suggested that individual participants with high knowledge but negative attitudes and intentions were less likely to undertake PSA testing.

### Strengths and Limitations of the Study

This is the first Web-based four-arm randomized controlled trial of informed decision making in PSA testing that has evaluated a Web-based PSA decision aid. The intervention has been shown to be associated with informed decision making and reduced PSA test uptake. The employment of an online questionnaire linked to a Web-based decision aid represents a departure from the traditional methods of evaluating decision aids where, normally, the participant would use the decision aid in a clinic setting, sometimes after seeing a clinician, and then be given a written questionnaire. A researcher would usually facilitate the whole process [10,21,22]. In contrast, participants in this trial used the decision aid and completed the online questionnaire in a setting of their choice, usually their own homes, with no training, thereby reducing researcher bias. In total, 514 participants were recruited for this study. This was less than the original recruitment target of 600 but sufficient to attain a power of 85% for the primary outcome, knowledge. Those men who did participate were mostly well educated and in the youngest of the three age groups, which limits the generalizability of the results. There was a significant loss to follow-up: 27% were lost between allocation and the first questionnaire despite use of a reminder questionnaire for those who initially failed to participate.

The intervention used in the study, Prosdex*,* had previously been the subject of a rigorous development process, which included field-testing [14,15], and the outcome measurements used in the questionnaire had previously been validated in other studies. The paper version of Prosdex contained the same textual information as the Web-based decision aid. However, participants in the paper version group retained their intervention while completing the questionnaire, unlike those in the Prosdex group who were led automatically from Prosdex to the online questionnaire and had no means of reviewing it while answering the questions. We have no means of ascertaining the impact of this potential contamination. Finally, the construction of the research website resulted in the baseline questionnaire being completed after viewing Prosdex, and it is possible that this may have had an effect on the impact of the intervention

### Comparison with Existing Literature

This trial builds on earlier studies that have considered the effect of PSA decision aids on the constituent components of informed decision making: knowledge, attitude, and behavior. Knowledge, as previously noted, has consistently been shown to improve with PSA decision aids [8], and recent studies have shown this to be the case with Web-based PSA decision aids [10,23]. With respect to attitude, Watson et al, in their trial of a paper-based PSA decision aid, found that their decision aid led to a less favorable attitude to PSA testing. Finally, in terms of behavior, we previously found in a systematic review of PSA decision aids that PSA testing decreased by 3.5% after using this aid [9]. This finding was reinforced in a more recent review by Volk and colleagues who found that men given a decision aid were less likely to take the PSA test (odds ratio 0.88) [8]. None of these studies, however, considered the three components of informed decision together as in our trial.

### Implications for Research and Practice

The controversy surrounding PSA testing has recently been reignited by two contrasting studies of prostate cancer screening, published simultaneously. First, a large European randomized study reported a 20% reduction in prostate cancer mortality. The authors estimated, however, that in order to prevent 1 death from prostate cancer, 1410 men would need to undergo PSA testing and 48 men would need to be treated for prostate cancer [24]. In the second study, another large randomized prostate cancer screening trial from the United States, no significant reduction in mortality was found after 7 to 10 years of follow-up [25]. Whatever the outcome of the screening debate, the fact remains that the PSA test has significant limitations and those men considering it will still require balanced information in order to make informed decisions. Moreover, the information will need to be easily and repeatedly accessible, as highlighted by the significant attrition in knowledge in this study after 6 months. The Web provides the ideal medium for this accessibility, and therefore, it is our opinion that it is not a question of whether Web-based PSA decision aids are required, but instead it is a question of how best to present the information to facilitate informed decision making.

This study has demonstrated that the Web-based PSA decision aid Prosdex has a positive impact on informed decision making in accordance with the UK Prostate Cancer Risk Management Strategy [2]. However, to maximize its impact and benefit the greatest number of men, its use needs to be promoted among the public as well as among health professionals. Prosdex requires an implementation strategy.

## Abbreviations

CONSORT: consolidated standards of reporting trials
DCS: Decisional Conflict Scale
GP: general practitioner
PCRMP: Prostate Cancer Risk Management Programme
PSA: prostate-specific antigen
RCT: randomized controlled trial
